# Examining the measurement of severity of intimate partner violence and its association to mental health outcomes: a narrative synthesis

**DOI:** 10.3389/fpubh.2024.1450680

**Published:** 2024-10-23

**Authors:** Sarah White, Lindsay Bearne, Angela Sweeney, Nadia Mantovani

**Affiliations:** ^1^St George’s School of Health and Medical Sciences, City St George’s, University of London, London, United Kingdom; ^2^Institute of Psychiatry, Psychology & Neuroscience, King’s College London, London, United Kingdom

**Keywords:** intimate partner violence (IPV), severity of IPV, measurement, mental health outcomes (MHO), narrative synthesis review

## Abstract

**Introduction:**

The aims of this synthesis were to investigate the relationship between IPV severity and mental health outcomes and shed light to gaps and limitations in existing methodologies used to assess IPV severity and its association with mental health outcomes.

**Methods:**

We conducted a two-stage narrative synthesis of 76 studies. First, we identified IPV measures used in at least five studies, focusing on their variations and severity score calculation. Then, we analyzed findings of studies correlating IPV severity with mental health outcomes, identifying features of measures and statistical methods influencing result consistency.

**Results:**

Measures of intimate partner violence were often modified from their original, potentially impact on the reliability and validity of these measures. The operationalization of violence severity varied across studies, leading to inconsistencies in scoring whereby compromising the consistency of severity levels across studies. We found lack of consistency in applying validated methods for scoring instruments to determine abuse severity. In this review, we consistently found that the severity of IPV and its various subtypes were linked to different mental health outcomes across multiple studies. We discovered evidence suggesting that experiencing more types of IPV was associated with worse mental health outcomes. Generally, higher levels of overall IPV severity and its specific subtypes were correlated with poorer mental health outcomes. However, our analyses did not reveal consistent patterns that would allow for a definitive determination of how individual IPV subtypes differently affect mental health outcomes. Nevertheless, we observed that increasing severity of physical IPV tended to have a notable impact on post-traumatic stress disorder (PTSD). Conversely, increasing severity of psychological IPV was consistently associated with depression. While sexual IPV severity was explored in fewer studies, the evidence regarding its impact on various mental health outcomes was less conclusive.

**Discussion:**

To achieve a comprehensive understanding of the mechanism by which IPV severity is related to mental health it may be time to take an alternative approach to measuring IPV severity. No IPV measures assessed the acceptability of the content to people who have experienced IPV. This is an important omission with significant consequences for the validity of the evidence base.

## 1 Introduction

Intimate partner violence (IPV) is a pervasive criminal justice, social and public health problem. It attracts attention in social and medical sciences, but accurate measurement is problematic and there is no comprehensive review of the ways in which IPV severity is measured. Without accurate, meaningful, and robust measurement of the severity of IPV, its deleterious impact on the mental health of survivors cannot be adequately examined. This study examined commonly utilized measures to assess the severity of IPV included in a recently published systematic review by White et al. (1). The examination focused on the scoring methods employed, the adaptation and calculation of severity scores, and explored how they impact the analysis of the relationship between the severity of IPV and mental health outcomes.

Intimate partner violence refers to behavior within a relationship that has physical, sexual and/or psychological impacts, and includes acts of physical aggression, sexual coercion, psychological abuse and controlling behaviors. This definition covers violence by both current and former spouses and partners (2). It is a multifaceted phenomenon that can manifest in a myriad of often co-occurring forms and is a gendered problem with women disproportionately impacted. Globally, an estimated 37% of women and girls aged 16 years or older have experienced lifetime physical, psychological, or sexual IPV, and an estimated 24% of women and girls aged 16 years or older have experienced IPV in the past year (1).

The experience of IPV is associated with a wide range of short-term and long-term physical and mental health sequelae, sexual and reproductive health problems, and death due to homicide and suicide (3–8). Although accurate screening for IPV should be a priority, currently screening for IPV is not integrated into any of the mental health risk assessment and management tools used in mental health services the UK (9). Additionally, IPV is rarely included as an exposure or an outcome in mental health research (2). Measuring IPV is a challenge as there is a lack of consensus on how types of IPV, which can vary by severity and frequency, combine into a pattern of behavior to represent an individual’s experience (2). In addition, the effect of abuse is cumulative with combined abuse, particularly abuse involving sexual IPV, being associated with the highest levels of harm, including risk of suicidal ideation and attempting suicide (10). Given the health, social, and economic costs of IPV, United Nations’ bodies, treaties, and declarations have called for better statistics on the nature, prevalence, causes, and consequences of violence against women as a basis for its elimination (11).

The assessment and measurement of IPV is controversial (12–16). Issues include the definition of violence, the boundary between violence and non-violent coercion (17). The assessment of repeated acts of IPV is contentious due to a lack of consensus on the measurement of IPV severity. The Conflict Tactics Scale (18), an early measure developed to study the prevalence and patterns of conflict within families, differentiates between minor and severe IPV, associating severe IPV with a higher likelihood of injury. However, this binary classification oversimplifies the complexity of IPV, as similar acts can have different consequences for male and female victims. Additional indicators of IPV severity include the frequency of incidents, the emotional impact, and resulting injuries (19–21). The problem with current severity operationalization is that it often overlooks these aspects simultaneously. Researchers have identified distinct types of IPV based on controlling behavior and employed cluster (22) or latent class analysis (23, 24) to identify severity classes, aiming to create mutually exclusive subgroups based on patterns of responses to observed categorical variables (24).

Walby et al. (15) suggest an IPV measurement framework that incorporates graded distinctions in the severity and frequency of violence and coercion and considers the consequences for victims. They acknowledge the temporal misalignment between perpetrator and victim in existing frameworks, where temporality is viewed as both episodic and continuous. Their proposal recognizes the duration of the action as repeated discrete incidents of violence, while the harm may manifest as a continuous state of fear. This challenges the assumption of alignment between one perpetrator, one victim, and one event, highlighting the accumulation of harm in high-frequency victims, particularly women (15, 16, 25).

Consensus is also lacking on the most accurate and psychometrically robust method for scoring behaviors in survey measures that assess abuse and violence. The legitimacy of using dichotomous splits to compare those experiencing abuse has been questioned, as it combines individuals with one incident with those experiencing frequent and severe abuse. Researchers emphasize the impact of decisions on scoring and classifying participants on their research results (13, 26, 27). Methodological questions have been raised about using unequal interval frequency categories, weighting items to improve sensitivity, and resolving identical scores produced through weighting, stemming from either a high frequency of mild incidents or a low frequency of severe incidents (28).

To adequately address the varied needs of those impacted by IPV, it is crucial to deepen our understanding of how the severity of such violence impacts mental health outcomes. While existing research indicates that women often endure more frequent and severe instances of IPV compared to men (17), precise measurement remains deficient. Yet, the measurement and analysis of IPV severity is complex, requiring careful consideration of population characteristics, methodological challenges and survivor involvement. Using data from studies investigating the association between severity of IPV and mental health outcomes included in the recently published systematic review, we addressed the research question: ‘With specific reference to the measurement of severity of violence what are the methodological challenges in examining the relationship between severity of IPV and mental health outcomes?’ The study objectives are:

1. To examine the commonly used measures of IPV severity and assess the different ways in which these were applied in practice.
2. To narratively review the evidence regarding the association between severity of IPV and mental health outcomes.
3. To provide recommendations on the development of new measures or amending old measures/approaches.

## 2 Materials and methods

### 2.1 Study design

This study adopted a narrative synthesis approach to explore a question that was not the primary focus of the initial research (1). In this context, we scrutinized the analysis of IPV severity across the studies we included and sought to understand how the severity of distinct forms of IPV related to various mental health outcomes.

The full details regarding the review search strategy, data sources and selection of the published review can be found in the aforementioned paper. To summarize, in the original review, full-text articles were evaluated against the following criteria: (a) those that included non-military women who were 16 years or older and were assessed for IPV experiences (overall, physical, psychological/emotional, and sexual) during their lifetime (lifetime IPV) or during the past year (i.e., 12 months prior to interview) using a validated IPV measure; (b) those which presented the results of peer-reviewed research based on quantitative methodology that provided mental health outcome data for at least one time point. The systematic review was registered on Prospero with the registration number CRD42020177744 (29).

All the 201 peer-reviewed studies that were included in the initial systematic review were searched to identify studies that used a tool to measure the severity of IPV either on a continuum or using an ordinal categorical format. This subset of studies was in the English language and published between 2012 and November 2020. The authors’ initial systematic review and meta-analysis expanded on an existing review by Trevillion, Oram, Feder, and Howard (30) that identified the prevalence of intimate partner violence in individuals diagnosed with a mental disorder. Hence, our initial review included a broader range of symptoms, issues, and needs related to mental illness diagnosis, which are well-documented as outcomes of exposure to IPV. As a result, we included more eligible studies compared to the aforementioned 2012 systematic review.

Downloaded full texts were evaluated against the following criteria: (a) those that included women and men who were 16 years or older and were assessed for severity of IPV (overall, physical, psychological/emotional, and sexual) during their lifetime or during the past year using a validated IPV measure; and/or (b) those which presented data on the association between severity of IPV and mental health outcomes for at least one time point.

### 2.2 Data extraction

Using a template designed and tested *a priori*, data extracted included: the settings, population sample, country, study design, IPV measure, type and timing of assessments, detail on how severity of IPV was measured (e.g., whether in a continuous form, categorical form, cumulative scores, or any other means to measure severity of IPV), and relevant findings regarding the association between IPV severity and MH outcomes. The range of statistics pertaining to the association between IPV severity and MH outcomes such as correlation coefficients (r), regression coefficients (b), odds ratios (OR) and adjusted odds ratios (AOR) were also extracted. When available 95% confidence intervals and *p*-values were also extracted. Where results were not tabulated or reported with appropriate statistics, verbatim text describing the findings of relevant analysis was extracted onto a bespoke data extraction tool.

### 2.3 Analysis

We conducted narrative analysis to synthesize our findings (31). In our initial synthesis, we focused on measures used in at least five studies, exploring how researchers employed IPV measures to calculate the severity of IPV. Specifically, we examined variations in their usage and severity score calculation, contrasting these practices with what was outlined in the measures’ development and validation papers. During this phase, we organized summaries of the studies, emphasizing the different types of severity scores used, laying the groundwork for the subsequent analysis.

In the next stage of our synthesis, we examined studies that reported the association between IPV severity and mental health outcomes. We systematically organized and tabulated these findings based on IPV severity type, mental health outcome, IPV measure, population under study, and the main results reported. The findings column in each Tables 2–5, details the extracted statistics from the analysis of the association. Adjusted results were tabulated whenever both adjusted and unadjusted analyses were reported. The synthesis aimed to identify where consistent evidence is present, assessing if type and severity of IPV is associated more or less with a specific mental health outcome and compare how findings are consistent across statistical methods.

## 3 Results

### 3.1 Description of the sample

From the original pool of 201 studies, 76 were included in this synthesis as theses measured the severity of IPV. Of these 76 the majority were conducted in the United States (*n* = 38) followed by Bangladesh (*n* = 3), Canada (*n* = 3), South Africa (*n* = 3), China (*n* = 3), Thailand (*n* = 3), Turkey (*n* = 3), Belgium (*n* = 2), Spain (*n* = 2), Brazil (*n* = 2), Vietnam (*n* = 2), Japan (*n* = 2), Australia (*n* = 1), Italy (*n* = 1), Cameroon (*n* = 1), Sweden (*n* = 1), United Kingdom (*n* = 1), Tanzania (*n* = 1), Lebanon (*n* = 1), Portugal (*n* = 1), and Greece (*n* = 1). One study was multi-site across different states: one in Baltimore, MD, USA, St. Croix and St. Thomas, U.S. Virgin Islands. Fifty-eight studies were located in high income countries, 11 in upper-middle countries, six in lower-middle countries, and one in low-income countries.

Twentynine studies recruited participants from the community (25 of which recruited women only) while 21 recruited IPV-exposed populations. Sixteen studies were with women in the perinatal period, while 10 recruited clinical-based samples (patients receiving healthcare at inpatient or outpatient or prison clinics unconnected to their experience of IPV.

### 3.2 Participants

Study sizes ranged from 14 to 14,575 participants, with a median of 303.5. Together, the studies included 54,131 participants (44,773 women; 9,349 men; 9 transgender).

### 3.3 Measurement of severity of IPV

Out of the original 201 studies, 76 (38%) measured the severity of intimate partner violence (IPV). Table 1 outlines the eight measures used in at least five studies, demonstrating the various ways that 62 (82%) of the included studies applied the measures and calculated IPV severity. Twenty-four studies (32%) calculated an overall IPV severity measure, 17 of these studies (22%) used a continuous scale, while six (8%) used a categorical variable. Forty-nine of these studies (64%) reported a measure of physical IPV severity, with 14 studies (18%) using a categorical variable, and 35 studies (46%) using a continuous scale. Psychological IPV severity was measured in 39 studies (51%), with 30 studies (39%) using a continuous scale and 9 (12%) using a categorical variable. Sexual IPV severity was reported by 27 studies (36%), with 20 studies (26%) using a continuous variable and 7 (9%) using a categorical one. One study (32) reported using an IPV measure, but no details were provided about how it was calculated.

**Table 1:** Scales used to measure severity of IPV, frequency of use, scoring methods, adaptation and type of variable.

Study	Type of IPV	Scoring methods	Continuous or categorical
Revised Conflict Tactics Scale (CTS2). # 34			
Wadji et al. (102)	Phys, Psych, Sexual	Tool used as intended, response format: Never = 0, once = 1, twice = 2, 3–5 times = 4, 6–10 times = 8, 11–20 times = 15, more than 20 times = 25	Continuous
Wong et al. (125)	Phys, Psych, Sexual		
Jaquier et al. (80)	Phys		
Hellmuth et al. (57)	Phys, Psych		
Fleming et al. (56)	Phys, Psych, Sexual		
Sullivan et al. (82)	Phys		
Young-Wolff et al. (51)	Phys	0, 1, 2, 4 = 3–5 times, 8 = 6–10 times, 11 = 10 or more times	Continuous
Reyes et al. (50)			Continuous
Flanagan et al. (49)			Continuous
Mertin et al. (122)	Overall^a^	Used 6-point scale (0 = this never happened to me; 6 = happened more than 20 times)	Continuous
Sullivan et al. (83)	Phys		
Yalch et al. (60)	Overall, Phys, Psych, Sexual		
Yalch et al. (59)	Overall		
Nathanson et al. (123)	Phys, Psych, Sexual		
Signorelli et al. (58)	Phys, Psych, Sexual	Used an 0–8 point scale to score IPV frequency for all items.	Continuous
Jeter et al. (126)	Phys, Psych	0 = never, 1 = rarely, 2 = sometimes, 3 = often, 4 = almost always	Continuous
Williams et al. (94)	Overall	0 = never, 1 = 1 time, 2 = twice, 3 = 3 or more times	Continuous
Wolford-Clevenger and Smith (53)	Phys	0 = no, 1 = yes was used to score all items. The subscale scores were the number of positively endorsed items within each subscale	Continuous
Norwood and Murphy (52)	Phys		
Hellemans et al. (77)	Phys	A 5-point Likert-type scale (0 = never to 4 = very often) was used on a single item from the physical assault subscale.	Continuous
Hellemans et al. (78)	Phys		
Tsai et al. (54)	Phys	Four items from the physical assault subscale were scoredon a four-point scale ranging from 1 (never) to 4 (many). Subsequently each item was standardized and the summary IPV index defined as the equally weighted average of the four z-scores.	Continuous
Sezgin and Punamäki (55)^b^	Phys, Psych, Sexual	Each item was scored as; 0 = never happened, 1 = not in the last year, but it did happen before, 2 = once, 3 = twice, 4 = 3–5 times, 5 = 6–10 times, 6 = 11–20 times in the past year, and 7 = more than 20 times; in the past year A principal component analysis was adopted to derive new subscales of IPV severity.	Continuous
Mugoya et al. (67)	Phys, Psych	Tool used as intended: Created a three level categorical variable for each IPV subtype; 0 = None, 1 = experienced minor acts only, 2 = experienced at least one severe act	Categorical
Kastello et al. (64)	Phys, Psych, Sexual		
Santos et al. (68)	Phys, Psych, Sexual		
Simmons et al. (69)	Phys		
Matseke et al. (66)	Phys	Reported levels of minor and severe physical IPV from reduced number of items of CTS2	
Kaplan et al. (63)	Phys		
Lysova et al. (24)	Phys		
Illangasekare et al. (62)	Overall	Created a three-level categorical variable, 1 = experience of no IPV or psychological IP; 2 = experience of minor physical or sexual IPV or 3 = experience of severe physical or sexual IPV in the past 6 months	Categorical
Lobato et al. (65)	Phys	The severity score used the 12 items as dichotomous and asked about victimization and perpetration of each event creating a score between 0 and 24. For analysis a three-level categorization was applied to the severity score: no event, a single event, and two or more events.	Categorical
Esie et al. (61)	Phys, Psych, Sexual	Psychological, physical, and sexual IPV was assessed at follow-up, using seven, 10, and, three items, respectively, taken from CTS2 and WHO. Responses were scored as 0 (one), 1–2 times (scored 1), 3–5 times (scored 2), 6–10 times (scored 3), greater than 10 times (scored 4). Each of these three IPV subtype scores was then categorized as “none” if women had not had recent exposure to IPV, or “low” “medium” or “high” based on tertiles of the non-zero values for each IPV severity score.	Categorical
Ziaei et al. (70)^c^	Overall	Used a 0–4 range to calculate the severity of IPV variable by summing up the different forms of IPV (physical, sexual, emotional, and controlling behavior) that an individual experienced.	Categorical
WHO Multi-Country Study on Women’s Health and Domestic Violence Against Women (WHO). # 10			
Gibbs et al. (109)	Overall, Phys, Psych, Sexual	Used the moderate and severe categories as intended but also reported whether a participant had experienced two or more types of IPV.	Categorical
Fisher et al. (72)	Overall, Phys		
Bernstein et al. (71)	Overall, Phys, Psych, Sexual		
Kapiga et al. (74)	Phys, Psych	The subscales had 6 and 4 items, respectively. Physical violence was considered severe if a participant reported having been hit, kicked, chocked or threatened with a weapon; and less severe if they reported having been pushed or slapped. For emotional abuse, severity was defined by the number of yes responses experienced by participant and analyzed as experienced, none, one event, or at least two events	Categorical
Esie et al. (61)	Phys, Psych, Sexual	Psychological, physical, and sexual IPV was assessed at follow-up, using seven, ten, and, three items, respectively, taken from CTS2 and WHO. Responses were scored as 0 (one), 1–2 times (scored 1), 3–5 times (scored 2), 6–10 times (scored 3), greater than 10 times (scored 4). Each of these three IPV subtype scores was then categorized as “none” if women had not had recent exposure to IPV, or “low” “medium” or “high” based on tertiles of the non-zero values for each IPV severity score.	Categorical
Tho Tran et al. (75)	Psych	Scored emotional violence (EV) as 0, 1, 2, 3+ types of EV, and 0, 1, 2–5, 5+ acts of EV	Categorical
Tran et al. (76)	Overall	Adopted a variable indicating whether someone had experienced all types of IPV (controlling, emotional, physical, and sexual).	Categorical
Hellemans et al. (77)	Psych	A 5-point Likert-type scale (0 = never to 4 = very often) was used on seven modified items. The severity score was computed by summing the scores to create severity score with the range 0–28	Continuous
Hellemans et al. (77)			
Xu et al. (79)	Overall	All items were scored as 0 = never, 1 = occasionally, 2 = sometimes, and 3 = often. Calculated three index scores of IPV severity: (i) an index of controlling behavior using three questions; (ii) an index of lifetime IPV victimization using four questions (both i and ii) scored as above and then averaged to produces scores between 0 and 3) and (iii) an index of total IPV victimization to approximate the severity of IPV victimization concomitantly constructed by averaging the two measures above.	Continuous
Psychological Maltreatment of Women Inventory. # 8			
Tirado-Muñoz et al. (84)	Psych	Adopted the original 58 item version.	Continuous
Sullivan et al. (82)		Used a 48-item version.	
Jaquier et al. (80)			
Sullivan et al. (83)		Adopted the short version PMWI-S scale.	
Saito et al. (81)			
Reyes et al. (50)		Added the PMWI-S six items to the verbal aggression items of the CTS2 and an item to assess stalking calculating a measure of psychological IPV severity as a sum of 21 items scored using the CTS2 response format	
Young-Wolff et al. (51)			
Flanagan et al. (49)		As above but one additional item which assess restriction of access to friends and family to produce measure of 22 items.	
Danger Assessment Scale (DAS). # 8			
Kamimura et al. (85)	Overall	Deployed the newer version of the tool with no deviation from the described scoring system.	Continuous
Peterson (89)			
Sabri et al. (90)			
Lucea et al. (87)			
Kulwicki et al. (86)		Deployed the newer version of the tool. They did not use the weighting to calculate the total score but summed up the number of affirmative responses.	
McFarlane et al. (88)		Deployed the newer version of the tool. They employed a weighted 19 item version but did not indicate which item was removed from the 20-item version.	
Peltzer et al. (91)	Overall	Removed one item dealing with sexual violence from the original 15-item DA scale and summed up the number of affirmative responses to produce a total score between 0 and 14. A low, medium, high categorization was used in analysis but was not defined.	Categorical
Kelly et al. (32)		DA was stated one of the measures in the study, but no information given as to how used	
Severity of Violence Against Women Scale (SVAW). # 8			
DeCou et al. (93)	Overall	Variations in the number of subscales utilized with participants’ responses being summed to yield a total score and included in their subsequent analyses.	Continuous
DeCou et al. (92)			
Kandeğer et al. (7)	Phys, Psych, Sexual	They pooled items across some subscales to produce threat, physical violence, and sexual violence subscales.	
McFarlane et al. (88)			
Sabri et al. (90)	Phys, Sexual	Only reported severity scores for physical and sexual abuse subscales.	
Lucea et al. (87)			
Peltzer et al. (91)	Phys, Psych, Sexual	Used the nine subscales of the SVAWS in parts of the analysis, but also combined subscales into physical, psychological, and sexual subscales.	
Saito et al. (81)	Phys, Sexual	Used the full SVAWS but only reported prevalence of varying severity of IPV and divided their sample into abused and non-abused groups.	
Sexual Experiences Survey (SES). # 8			
Williams et al. (94)	Sexual	Used the SES-SFV version, though they did not assign participants to an ordinal category as required, but rather summed up the items to calculate a total sexual IPV severity score.	Continuous
Young-Wolff et al. (51)			
Reyes et al. (50)		Replaced the yes/no response format of the SES with the CTS2 response form. Summed up the items to calculate a total sexual IPV severity score.	
Sullivan et al. (83)			
Jaquier et al. (80)			
Sullivan et al. (82)			
Norwood et al. (52)		Replaced the yes/no response format of the SES with the CTS2 response form. Combined the SES and sexual coercion subscale of the CTS2 and applied exploratory factor analysis to identify a two-factor solution, six items reflecting sexual violence and seven items reflecting sexual coercion.	
Flanagan et al. (49)		Replaced the yes/no response format of the SES with the CTS2 response form. To overcome the excessive skew after summing the items recoded sexual IPV into an ordinal variable (0 = no victimization, 1 = moderate sexual victimization, and 2 = sexual victimization with penetration).	Categorical
Composite Abuse Scale (CAS). # 5			
Tutty et al. (101)	Overall, Phys, Psych	All used the original CAS, scored and analyzed the scale as described by the original authors.	Continuous
Ferrari et al. (100)	Overall, Phys, Psych		
Khadra et al. (96)	Phys		
Edmond et al. (124)	Overall, Phys, Psych		
Daugherty et al. (6)	Overall	Used the CAS-SF.	
Index of Spousal Abuse. # 5			
Kita et al. (98)	Phys, Psych	Used as authors intended.	Continuous
Watson-Singleton et al. (99)			
Peterson (89)			
Comeau and Davies (97)			
Kelly and Pich (32)		Used clinical cut-offs as an inclusion criterion rather than a variable for statistical analysis.	

a The nine-item violence subscale of the Conflict Tactics Scale (33) was extended to an 18-item measure in order to assess additional factors of IPV, including verbal, sexual, and financial abuse (127).

b Used short form of CTS2 [CTS2S; (128)].

c Used short form of CTS2 [CTS2S; (128)] in combination with WHO tool to produce a modified scale.

## 4 Findings

### 4.1 Measures of intimate partner violence

Twenty-two different measures of IPV were used across the 76 studies in our sample. Thirteen studies (17%) utilized two IPV measures, and nine studies (12%) used three IPV measures. The Revised Conflict Tactics Scale (CTS2) (18, 33, 34) was the most frequently used measure, with 35 studies (45%) employing it to measure at least one type of IPV. Ten studies (13%) used the WHO standardized questionnaire (35, 36). Eight studies (10%) employed the Danger Assessment scale (DA) (37–39), the Psychological Maltreatment of Women Inventory (PMWI) (40, 41), the Sexual Experiences Survey (SES) (42–45), and the Severity of Violence Against Women Scale (SVAW) (46). The Composite Abuse Scale (CAS) by Hegarty et al. (47) and the Index of Spousal Abuse by Hudson and McIntosh (48) were used in five studies (6%). Of the remaining 14 scales that were utilized, two were used three times (Abuse Assessment Screen; Abuse Behavior Inventory), three were used twice (Domestic Violence Scale; Woman abuse screening measure; Women’s Experiences of Battering) and the remaining nine used just once (Cumulative trauma experiences; CVES Research Version; Measure of Psychologically Abusive Behaviors; Multidimensional measure of emotional abuse; Potentially Harmful Behavior Scale; Pregnancy Risk Assessment Monitoring System; Trauma History Questionnaire; NorVold Abuse Questionnaire; Behavioral Risk Factor Surveillance System). In six studies, the PMWI was used to measure psychological IPV alongside the CTS2 which measured physical IPV, and the SES which measured sexual IPV (Table 1).

Eight measures (CTS2, WHO, PMWI, DA, SVAW, SES, CAS, ISA) were employed by at least five of the included studies (see Table 1). None of the measures that were modified by the researchers were revalidated prior to their use.

#### 4.1.1 Revised conflict tactics scale

Data collected using the CTS2 can be reported as prevalence, chronicity and severity of IPV [for descriptions see (18, 34)]. Chronicity for individuals with at least one experience of violence in a subscale is scored based on the frequency. Scores are summed for a continuous chronicity/severity score. In contrast severity, excluding negotiation, categorizes acts into minor or severe, with respondents classified by severity: severe (at least one severe act), minor (at least one minor act but no severe act), and none (no reported acts).

Studies measured IPV severity on a continuum and/or using a categorical variable. Of the 34 studies using the CTS2, 23 reported either descriptive and/or analytical statistics with a continuous variable of IPV chronicity, intensity, or severity (Table 1). Researchers did not consistently adhere to a validated structure and scoring scheme: seventeen studies had variations in how scales were truncated or extended, response formats altered, or scores calculated. Some studies did not use the highest frequency category (49–51) but retained the weighted scores. In contrast, two studies scored all items using 0 = no, 1 = yes, summing the items so that the subscale scores were the number of positively endorsed items within each subscale (52, 53). Two studies did not sum all items within subscales. Tsai, Tomlinson, Comulada, and Rotheram-Borus (54) used four items from the physical assault subscale (CTS2) scoring responses on a four-point scale ranging from 1 (never) to 4 (many). Each item was standardized and the summary IPV index defined as the equally weighted average of the four z-scores. Sezgin and Punamäki (55) adopted principal component analysis to derive IPV severity subscales. In five studies, severity scores were calculated for respondents who had not experienced at least one act of IPV, contradicting guidance (56–60).

Eleven studies reported IPV severity using a categorical form (24, 61–70), with eight studies using the recommended labels of minor and severe (33). Three studies used different approaches to create a categorical severity score. Esie et al. (61) developed three composite scores of IPV severity by combining items from the CTS2 and the WHO questionnaire. The frequency of psychological, physical, and sexual IPV was recorded as never (scored 0) as 1–2 times (scored 1), 3–5 times (scored 2), 6–10 times (scored 3), greater than 10 times (scored 4). Item scores were then summed to create a severity score. Each of these three scores was categorized as “none” “low” “medium” or “high” based on tertiles of the non-zero values for each IPV severity score. Lobato et al. (65) applied a three-level categorization to the composite score to use in analysis: no event, a single event, and two or more events. Ziaei et al. (70) used a 0–4 labeled categorical variable to calculate the severity of IPV by summing the different forms of IPV (physical, sexual, emotional, and controlling behavior) that an individual experienced.

#### 4.1.2 WHO multi-country study on women’s health and domestic violence against women

Data collected using this measure can be reported as prevalence of physical and sexual IPV against women and its correlation with health outcomes in culturally diverse countries. The severity of a physically violent act is ranked according to its likelihood of causing physical injuries and defined dichotomously (moderate or severe) [see (35)].

Of the 10 studies using this measure, seven studies created categorical ratings of IPV severity (61, 71–76), with four studies employing the minor and severe category ratings to do so. Esie et al. (61) combined items from the WHO and CTS2 as described above to produce a four-level variable. Tran et al. (76) calculated a binary variable indicating whether someone had experienced all types of IPV (controlling, emotional, physical, and sexual). Tho Tran et al. (75) scored emotional violence (EV) as 0, 1, 2, 3+ types of EV, and 0, 1, 2–5, 5+ acts of EV. The remaining two studies (77, 78) created a continuous psychological IPV severity variable by applying a 5-point Likert-type scale (0 = never to 4 = very often) on seven modified items, the severity score computed by summing the scores, range 0–28. Xu et al. (79) calculated three continuous index scores of IPV severity.

#### 4.1.3 Psychological maltreatment of women inventory

This measure assesses nonphysical abusive behavior in male IPV perpetrators with responses being rated on a Likert-style scale (1 = never to 5 = very frequently). Scores are calculated by summing items within each subscale. A shorter 14-item version, PMWI-S, maintains these subscales (41).

Eight studies used this measure and produced continuous measures of the severity of psychological IPV by summing the item scores (49–51, 80–84). Only one study used the original 58-item measure (84), while the remaining studies either used the PMWI-S and adopted the intended response format, or added to the PMWI-S six items from CTS2 and used the CTS2 response format.

#### 4.1.4 Danger assessment scale

This measure assesses the likelihood of lethality or near lethality in cases of IPV. The revised version (38) defined danger levels such as variable danger (0–7), increased danger (9–13), severe danger (14–17), and extreme danger (18 and above). This measure was adopted in eight studies, of which six produced continuous measures of the severity of IPV (risk of lethality) by summing the item scores (85–90) and two reported IPV severity using a categorical form (32, 91). Six studies used the newer version 20 item scale although one study dropped an item (88). Kulwicki et al. (86) and Peltzer and Pengpid (91) created categorical ratings of IPV severity. While the former did not use the weighting to calculate the total score but summed up the number of affirmative responses, the latter removed one item dealing with sexual violence from the original 15-item DA scale and summed up the number of affirmative responses to produce a total score between 0 and 14. A low, medium, high categorization was used in the analysis but was not defined.

#### 4.1.5 Severity of violence against women scale

The SVAW assesses the frequency and severity of physical aggression, allowing researchers to explore different severity levels and analyze the distinct effects of various violence types. It is comprised of nine subscales measuring two major dimensions (threats and actual violence).

This measure was adopted in eight studies all of which produced continuous measures of the severity of IPV by summing the item scores (7, 81, 87, 88, 90–93). There were variations in the number of subscales used, for example, DeCou et al. (92), summed participants’ responses to yield a total IPV severity score, which was included in their subsequent analyses. The remaining studies reported the subscales. Saito et al. (81) used the full SVAWS but only reported prevalence of varying severity of IPV and divided their sample into abused and non-abused groups.

#### 4.1.6 Sexual experiences survey

The SES assesses various sexual victimization experiences through 10 behaviorally specific items, covering unwanted and non-consensual encounters, including sexual coercion, attempted rape, and rape. The SES is scored on an objective severity continuum, with rape assigned a score of 4, attempted rape a score of 3, coercion a score of 2, contact a score of 1, and no victimization a score of 0. The SES was later revised to create the Short Form Victimization (SES-SFV) (42).

Eight studies used the SES, of which seven created continuous IPV severity variables (50–52, 80, 82, 83, 94), and one created categorical ratings of IPV severity (49). None of the studies used the objective severity outcome as defined by the authors. Williams et al. (94) used the SES-SFV version but summed up the items to calculate a sexual IPV severity score. The remaining studies all replaced the yes/no response format of the SES with the CTS2 response form. Four studies (50, 80, 82, 83) summed up the items to calculate a total sexual IPV severity score. To overcome the excessive skew after summing the items as intended, Flanagan et al. (49) recoded sexual IPV into an ordinal variable (0 = no victimization, 1 = moderate sexual victimization, and 2 = sexual victimization with penetration).

#### 4.1.7 Composite abuse scale

The CAS is a comprehensive abuse measure with four dimensions: severe combined abuse, emotional abuse, physical abuse, and harassment. A 15-item version (CAS Short Form, CASR-SF) was later created, covering physical, sexual, and psychological abuse, with scores ranging from 0 to 75. The total score, calculated as the mean of responses multiplied by 15, is recommended over subscale scores (95).

Five studies used CAS and reported severity on a continuous IPV severity variable. Daugherty et al. (6), however, used the CAS-SF, and Khadra et al. (96) used only the Physical Abuse subscale. The remaining studies used the original CAS, and scored and analyzed this measure as described by the original authors.

#### 4.1.8 Index of spousal abuse

The ISA measures the severity of physical and non-physical aggression (referred to in this paper as psychological for consistency) by an intimate partner, derived from the CTS. Each item is rated from 1 (never) to 5 (very frequently). Subscale scores, ranging from 0 to 100, are calculated with weighted items, giving greater importance to more serious forms of abuse. Clinical cut-offs are set at 10 for ISA-P (physical) and/or 25 for ISA-NP (non-physical), identifying individuals likely experiencing spousal abuse.

Five studies used this measure as intended, creating continuous IPV severity variables (32, 89, 97–99). The study by Kelly and Pich (32) used its clinical cut-offs as an inclusion criterion rather than a variable for statistical analysis.

### 4.2 Analyzing the association between severity of IPV and mental health outcomes

Tables 2–5, highlight the studies that explored the association between severity of IPV, either overall or by subtype, and mental health outcomes. In each table significant associations have been highlighted in bold.

**Table 2:** Association between severity of overall IPV and mental health outcomes.

MHO	Study	Population F-female, M-male	Tool	Variable type	Findings
Depression	Edmond et al. (124)	IPV exposed (F)	CAS	Con	“There were no differences between those who were experiencing PTSD and/or depression and those who were not in terms of the severity or type of IPV that had been experienced in the previous 12 months.” No figures reported
Depression	Ferrari et al. (100)	IPV exposed (F)	CAS	Con	AOR = 1.03 (95% CI:0.99, 1.05)
Depression	Tutty et al. (101)	IPV exposed (F)	CAS	Con	**“Correlations between the mental health scales and the CAS-Total were numerically lower (r’s ranging from 0.14 to 0.28) but still statistically significantly related (*p*s of 0.01).”**
Depression	Daugherty et al. (6)	IPV exposed (F)	CAS-SF	Con	*r* = 0.15, *p* > 0.05
Depression	Mertin et al. (122)	IPV exposed (F)	CTS2	Con	***r* = 0.221, *p* < 0.05**
Depression	Sezgin and Punamäki (55)	Perinatal	CTS2	Con	***b* = 0.21, *p* < 0.0001**
Depression	Tsai et al. (54)	Perinatal	CTS2	Con	***b* = 1.04; (95% CI, 0.61–1.47)**
Depression	Williams et al. (94)	IPV exposed (F)	CTS2	Con	***r* = 0.219, *p* < 0.01**
Depression	Illangasekare et al. (62)	IPV exposed (F)	CTS2	Cat	Minor physical or sexual IPV only vs. none AOR = 3.17 (95% CI 0.65, 15.5) *p* = 0.154; **Severe physical or sexual IPV *vs* none AOR = 5.34 (95% CI 1.53, 18.6) *p* = 0.009**
Depression	Mugoya et al. (67)	Community (F)	CTS2	Cat	Minor AOR = 0.95 (95% CI 0.60, 1.49); **Severe AOR = 2.02 (95% CI 1.26, 3.24); Very severe AOR = 2.84 (95% CI 1.75, 4.62)**
Depression	Simmons et al. (69)	Community (M/F)	CTS2	Cat	Females - Minor OR = 0.96, *p* = 0.910; Severe OR = 2.72, *p* = 0.060 Males - Minor b = −0.34, *p* = 0.550; Severe b = 0.73, *p* = 0.220
Depression	Peterson (89)	IPV exposed (F)	DAS	Con	**“Women with depression symptoms scored significantly higher on the DA than the group of women without depression [*t*(1, 40) = −2.399, *p* < 0.01].”**
Depression	Kulwicki et al. (86)	Community (F)	DAS	Con	***r* = 0.44, *p* < 0.001**
Depression	Peltzer et al. (91)	IPV exposed (F)	DAS	Con - Cat	***r* = 0.33, *p* < 0.01.** High danger AOR 2.44 (0.89, 5.45), *p* > 0.05
Depression	Comeau et al. (97)	IPV exposed (F)	ISA	Con	“Patterns of IPV severity suggest that although more severe abuse experiences are associated with depressive symptoms, they may not translate into depression diagnoses”
Depression	Xu et al. (79)	Community (M/F)	WHO	Con	**Women *b* = 0.284, *p* < 0.001; Men *b* = 0.267, *p* < 0.001**
Depression	Gibbs et al. (109)	Informal settlements (F)	WHO	Cat	“As with depressive symptoms, the highest prevalence of suicidal ideation in all combinations was where physical or sexual IPV was combined with emotional or economic IPV.”
PTSD	Edmond et al. (124)	IPV exposed (F)	CAS	Con	“There were no differences between those who were experiencing PTSD and/or depression and those who were not in terms of the severity or type of IPV that had been experienced in the previous 12 months.” No figures reported
PTSD	Ferrari et al. (100)	IPV exposed (F)	CAS	Con	**AOR = 1.03 (95% CI:1.03, 1.04)**
PTSD	Tutty et al. (101)	IPV exposed (F)	CAS	Con	**“Correlations between the mental health scales and the CAS-Total were numerically lower (r’s ranging from 0.14 to 0.28) but still statistically significantly related (ps of 0.01).”**
PTSD	Daugherty et al. (6)	IPV exposed (F)	CAS-SF	Con	***r* = 0.23, *p* < 0.05**
PTSD	Williams et al. (94)	IPV exposed (F)	CTS2	Con	***r* = 0.247, *p* < 0.01**
Trauma symptoms	Yalch et al. (60)	Community (F)	CTS2	Con	***r* = 0.25, *p* < 0.05**
PTSD	Sabri et al. (90)	IPV exposed (F)	DAS	Con	**“Women with co-occurring PTSD and depression problems had significantly higher mean scores on the danger assessment than did women in the depression-only or the neither PTSD nor depression problems group (*p* < 0.05).”** **“After controlling for sociodemographic variables, injuries, and severity of IPV, risk for lethality was not a significant predictor of co-occurring PTSD and depression for any [ethnic] group”**
PTSD	Peterson (89)	IPV exposed (F)	DAS	Con	**“Women with PTSD scored significantly higher on the DA than the group of women without PTSD [*t*(1,40) = −2.91, *p* < 0.01].**
PTSD	DeCou et al. (92)	IPV exposed (F)	SVAW	Con	**“(Partner violence) PV (*β* = 0.22, *t* = 3.15, *p* = 0.002), and a PV × DVCSE (Domestic Violence Coping Self-Efficacy) (β = −0.54, *t* = −2.04, *p* = 0.044) interaction term emerged as significant independent variables associated with PTSD scores, *F*(5, 96) = 12.10, p < 0.001”**
PTSD	DeCou et al. (93)	IPV exposed (F)	SVAW	Con	***r* = 0.29, *p* < 0.001**
Anxiety	Ferrari et al. (100)	IPV exposed (F)	CAS	Con	**AOR = 1.03 (95% CI:1.01, 1.05)**
Anxiety	Daugherty et al. (6)	IPV exposed (F)	CAS-SF	Con	*r* = 0.09, *p* > 0.05
Anxiety	Mertin et al. (122)	IPV exposed (F)	CTS2	Con	***r* = 0.420, *p* < 0.01**
Anxiety	Sezgin and Punamäki (55)	Perinatal	CTS2	Con	***b* = 0.21, *p* < 0.0001**
Psychological distress	Tutty et al. (101)	IPV exposed (F)	CAS	Con	**“Correlations between the mental health scales and the CAS-Total were numerically lower (r’s ranging from 0.14 to 0.28) but still statistically significantly related (ps of 0.01).”**
Psychological distress	Ziaei et al. (70)	Perinatal	CTS2	Cat	**Cumulative number of different forms of DV: 1 – AOR = 1.90 (95% CI 1.58, 2.30); 2 – AOR = 3.89 (95% CI 3.08, 4.70); 3 – AOR = 5.31 (95% CI 4.15, 6.80); 4 – AOR = 8.79 (95% CI 6.26, 12.34)**
Psychological distress	Kamimura et al. (85)	IPV exposed (F)	CTS2	Cat	“We compared the means of the health outcome variables by the Danger Assessment severity scores, but no difference was found.”
Opioid use	Williams et al. (94)	IPV exposed (F)	CTS2	Con	***r* = 0.317, *p* < 0.01**
Alcohol use	Yalch et al. (59)	Community (F)	CTS2	Con	***r* = 0.15, *p* < 0.05**
Suicidal behavior	Peltzer et al. (91)	IPV exposed (F)	DAS	Con - Cat	***r* = 0.54, *p* < 0.01. High danger AOR 63.17 (11.32, 352.59), *p* < 0.001**
Suicidal ideation	Gibbs et al. (109)	Informal settlements (F)	WHO	Cat	“As with depressive symptoms, the highest prevalence of suicidal ideation in all combinations was where physical or sexual IPV was combined with emotional or economic IPV.”
Common mental disorder (CMD)	Fisher et al. (72)	Perinatal	WHO	Cat	**Lifetime IPV; One type of violence AN CMD 2.3 (1.4–4.1) PN CMD 1.9 (1.1–3.5);** **Two or three types AN CMD 2.6 (1.3–5.3) PN CMD 4.3 (2.2–8.6)** **Postpartum IPV; One type of violence PN CMD 5.0 (1.6–15.7); Two or three types PN CMD10.1 (2.8–37.3)**
Common mental disorder (CMD)	Tran et al. (76)	Perinatal	WHO	Cat	**All types of violence; AOR = 2.31 (1.32, 4.02)**

r, correlation coefficient; b, regression coefficient; AOR, adjusted odds ratio; p: *p*-value; OR, odds ratio; CI, confidence intervals. Variable type, continuous, categorical.

**Table 3:** Association between severity of physical IPV and mental health outcomes.

MHO	Study	Population F-female, M-male	Tool	Variable type	Findings
Depression	Signorelli et al. (58)	Help-seeking (F)	CTS2	Con	*b* = 0.069, *p* = 0.609
Depression	Sullivan et al. (82)	IPV exposed (F)	CTS2	Con	***r* = 0.35, *p* < 0.01**
Depression	Wadji et al. (102)	IPV exposed (F)	CTS2	Con	***r* = 0.355, *p* = 0.031**
Depression	Wolford-Clevenger et al. (53)	IPV exposed (F)	CTS2	Con	*r* = 0.09, *p* > 0.05
Depression	Flanagan et al. (49)	IPV exposed (F)	CTS2	Con	***r* = 0.22, *p* < 0.01**
Depression	Hellmuth et al. (57)	Perinatal	CTS2	Con	*r* = 0.08, *p* > 0.05
Depression	Nathanson et al. (123)	IPV exposed (F)	CTS2	Con	*r* = 0.04, *p* > 0.05
Depression	Esie et al. (61)	Community (F)	CTS2	Cat	None AOR = 1; Low 1.01 (0.80–1.28); **Medium 1.52 (1.09–2.12); High 2.44 (1.94–3.08)**
Depression: Antenatal Postnatal	Kita et al. (98)	Perinatal	ISA	Con	***r* = 0.13, *p* < 0.01** *r* = 0.07, *p* > 0.05
Postnatal Depression	Lobato et al. (65)	Perinatal	SVAW	Cat	“Among women with alcohol positive partners, while a single act of physical IPV during pregnancy failed to show any bearing with PPD**, the occurrence of two or more events increased the chance by almost fourfold**. For women whose partners did not misuse alcohol, although, the relationship between physical IPV and PPD showed a different pattern. **Although a single episode of physical IPV was significantly associated with PPD**, **the effect of two or more events was only statistically marginal in the final model**.”
Depression	Mugoya et al. (67)	Community (F)	SVAW	Cat	**Minor AOR = 1.69 (95% CI 1.12, 2.55); Severe AOR = 2.92 (95% CI 1.94, 4.40)**
Depression	Peltzer et al. (91)	IPV exposed (F)	SVAW	Con - Cat	***r* = 0.29, *p* < 0.01:** Mild AOR 0.48 (95% CI 0.20, 1.24) Minor 1.31 (95% CI 0.50, 3.40) Moderate 1.67 (95% CI 0.60, 4.66), Severe 1.95 (95% CI 0.81, 4.72)
Depression	Xu, X. et al. (79)	Community (M/F)	WHO	Con	**Women *b* = 0.219, *p* < 0.001; Men *b* = 0.218, *p* < 0.001**
PTSD	Khadra et al. (96)	IPV exposed (F)	CAS	Con	***r* = 0.719, *p* < 0.05**
PTSD	Sullivan et al. (82)	IPV exposed (F)	CTS2	Con	***r* = 0.54, *p* < 0.01**
PTSD	Wolford-Clevenger et al. (53)	IPV exposed (F)	CTS2	Con	***r* = 0.32, *p* < 0.01**
PTSD	Flanagan et al. (49)	IPV exposed (F)	CTS2	Con	***r* = 0.41, *p* < 0.01**
PTSD	Jeter et al. (126)	Community (F)	CTS2	Con	*b* = 0.08, *p* > 0.05
PTSD	Nathanson et al. (123)	IPV exposed (F)	CTS2	Con	*r* = 0.17, *p* > 0.05
Trauma symptoms	Yalch et al. (60)	Community (F)	CTS2	Con	***r* = 0.25, *p* < 0.05**
PTSD	Norwood et al. (52)	IPV exposed (F)	CTS2, SES	Con	***r* = 0.27, *p* < 0.001**
PTSD	Kastello et al. (64)	IPV exposed (F)	SVAW	Cat	No association between categorical severity physical IPV and PTSD, *p* = 0.807
PTSD	Sabri et al. (90)	IPV exposed (F)	SVAW	Con	**“Women with co-occurring PTSD and depression problems had higher mean scores on severity of physical abuse than did women with depression-only or PTSD only problem (*p* < 0.05).”**
Anxiety	Wadji et al. (102)	IPV exposed (F)	CTS2	Con	***r* = 0.430, *p* = 0.011**
Antenatal anxiety Postnatal anxiety	Kita et al. (98)	Perinatal	ISA	Con	***r* = 0.12, *p* < 0.01** ***r* = 0.14, *p* < 0.01**
Psychological distress Change in distress	Kaplan et al. (63)	Community (F)	CTS2	Cat	**Minor b = 0.09 (se = 0.03), *p* < 0.01;** Severe *b* = −0.04 (se = 0.04), *p* > 0.05 Minor *b* = −0.02 (se = 0.03), Severe *b* = −0.01 (se = 0.03), both *p* > 0.05, respectively
Psychological distress	Hellemans et al. (77)	Community (M/F)	CTS2	Con	*r* = 0.06, *p* > 0.05
Psychological distress	Hellemans et al. (77)	Community (M/F)	CTS2	Con	***r* = 0.16, *p* < 0.01**
Psychological distress	Ziaei et al. (70)	Perinatal	SVAW	Cat	**Moderate AOR = 2.41 (95% CI 2.03, 2.87); Severe AOR = 3.25 (95% CI 2.50, 4.22)**
Drug misuse	Reyes et al. (50)	IPV exposed (F)	CTS2	Con	***r* = 0.36, *p* < 0.01**
Drug misuse	Flanagan et al. (49)	IPV exposed (F)	CTS2	Con	***r* = 0.21, *p* < 0.01**
Drug misuse	Nathanson et al. (123)	IPV exposed (F)	CTS2	Con	*r* = −0.03, *p* > 0.05
Alcohol misuse	Reyes et al. (50)	IPV exposed (F)	CTS2	Con	***r* = 0.36, *p* < 0.01**
Alcohol related problems Alcohol dependence	Sullivan et al. (82)	IPV exposed (F)	CTS2	Con	***r* = 0.41, *p* < 0.01** AOR = 1.25, *p* > 0.05
Alcohol misuse	Flanagan et al. (49)	IPV exposed (F)	CTS2	Con	***r* = 0.14, *p* < 0.01**
Alcohol misuse	Hellmuth et al. (57)	Perinatal	CTS2	Con	*r* = −0.03, *p* > 0.05
Alcohol misuse	Nathanson et al. (123)	IPV exposed (F)	CTS2	Con	*r* = −0.04, *p* > 0.05
Alcohol misuse	Watson-Singleton et al. (99)	IPV exposed (F)	ISA	Con	***r* = 0.17, *p* = 0.030**
Suicidal behavior	Peltzer et al. (91)	IPV exposed (F)	SVAW	Con - Cat	***r* = 0.28, *p* < 0.01:** Mild AOR 2.64 (95% CI 0.60, 11.64) Minor 0.34 (95% CI 0.05, 2.14) Moderate 1.96 (95% CI 0.42, 9.23) Severe 0.49 (95% CI 0.08, 2.98)
Suicidal ideation	Wolford-Clevenger et al. (53)	IPV exposed (F)	CTS2	Con	*r* = 0.08, *p* > 0.05
Suicidal ideation	Kandeg Kandeğer et al. (7)	IPV exposed (F)	SVAW	Con	***r* = 0.51, *p* < 0.01**
Deliberate self-harm (DSH)	Jaquier et al. (80)	IPV exposed (F)	CTS2	Con	Not significant in linear discriminant function
CMD: Depressed anxious mood, Depressive thoughts	Santos et al. (68)	Community (F)	SVAW	Cat	**Minor OR = 3.07 (95% CI 1.29; 10.63);** Severe OR = 2.07 (95% CI 0.61; 7.09) **Minor OR = 5.92 (95% CI 3.22; 10.87); Severe OR = 7.03 (95% CI 3.05; 17.24)**

r, correlation coefficient; b, regression coefficient; AOR, adjusted odds ratio; p, *p*-value; OR, odds ratio; CI, confidence intervals. Variable type, continuous, categorical.

**Table 4:** Association between severity of psychological IPV and mental health outcomes.

MHO	Study	Population F-female, M-male	Tool	Variable type	Findings
Depression	Hellmuth et al. (57)	Perinatal	CTS2	Con	***r* = 0.32, *p* < 0.01**
Depression	Nathanson et al. (123)	IPV exposed (F)	CTS2	Con	***r* = 0.28, *p* < 0.01**
Depression	Signorelli et al. (58)	Help-seeking (F)	CTS2	Con	*b* = 0.090, *p* = 507
Depression	Wadji et al. (102)	IPV exposed (F)	CTS2	Con	Non-significant, r’s not reported
Depression	Mugoya et al. (67)	Community (F)	CTS2	Cat	Minor AOR = 1.00 (95% CI 0.64, 1.56); **Severe AOR = 2.25 (95% CI 1.49, 3.40)**
Depression	Flanagan et al. (49)	IPV exposed (F)	CTS2/PMWI	Con	***r* = 0.28, *p* < 0.01**
Depression	Esie et al. (61)	Community (F)	CTS2 WHO	Cat	None AOR = 1; Low 0.80 (0.60–1.05); Medium 1.31 (0.89–1.91); **High 2.27 (1.62–3.17)**
Depression: Antenatal Postnatal	Kita et al. (98)	Perinatal	ISA	Con	***r* = 0.22, *p* < 0.001** ***r* = 0.18, *p* < 0.001**
Depression	Sullivan et al. (82)	IPV exposed (F)	PMWI	Con	***r* = 0.46, *p* < 0.01**
Depression	Peltzer et al. (91)	IPV exposed (F)	SVAW	Con – Cat	***r* = 0.44, *p* < 0.01:** Symbolic AOR 1.14 (95% CI 0.51, 4.07) Mild 1.87 (95% CI 0.85, 4.12) Moderate 1.04 (95% CI 0.48, 2.81), Severe 2.40 (95% CI 0.97, 5.91)
Depression	Xu et al. (79)	Community (M/F)	WHO	Con	**Women *b* = 0.095, *p* < 0.001; Men *b* = 0.064, *p* < 0.001**
Depression	Tho Tran et al. (75)	Perinatal	WHO	Cat	**Not exposed AOR = 1; One type of emotional violence 2.28 (1.35–3.86); Two type of emotional violence 3.15 (1.17–8.51);** Three or more types of emotional violence and above 3.16 (0.83–12.03)
PTSD	Nathanson et al. (123)	IPV exposed (F)	CTS2	Con	***r* = 0.22, *p* < 0.05**
Trauma symptoms	Yalch et al. (60)	Community (F)	CTS2	Con	***r* = 0.26, *p* < 0.05**
PTSD	Flanagan et al. (49)	IPV exposed (F)	CTS2/PMWI	Con	***r* = 0.46, *p* < 0.01**
PTSD	Kastello et al. (64)	IPV exposed (F)	CTS2/PMWI	Cat	No association between categorical severity psychological IPV and PTSD, *p* = 0.797
PTSD	Norwood et al. (52)	IPV exposed (F)	MMEA	Con	***r* = 0.47, *p* < 0.001**
PTSD	Jeter et al. (126)	Community (F)	MPAB	Con	***b* = 0.30, *p* < 0.001**
PTSD	Sullivan et al. (82)	IPV exposed (F)	PMWI	Con	***r* = 0.56, *p* < 0.01**
PTSD	Sabri et al. (90)	IPV exposed (F)	WEB	Con	**“The co-occurring problems group had significantly higher scores on psychological abuse compared to women with depression-only problems (*p* < 0.05).”**
Anxiety	Wadji et al. (102)	IPV exposed (F)	CTS2	Con	Non-significant, r’s not reported
Anxiety: Antenatal Postnatal	Kita et al. (98)	Perinatal	ISA	Con	***r* = 0.22, *p* < 0.001** ***r* = 0.24, *p* < 0.001**
Psychological distress	Hellemans et al. (77)	Community (M/F)	WHO	Con	***r* = 0.19, *p* < 0.01**
Psychological distress	Hellemans et al. (78)	Community (M/F)	WHO	Con	***r* = 0.19, *p* < 0.01**
Drug misuse	Nathanson et al. (123)	IPV exposed (F)	CTS2	Con	*r* = −0.05, *p* > 0.05
Drug misuse	Flanagan et al. (49)	IPV exposed (F)	CTS2/PMWI	Con	***r* = 0.11, *p* < 0.05**
Drug misuse	Reyes et al. (50)	IPV exposed (F)	PMWI	Con	*r* = 0.15, *p* > 0.05
Alcohol misuse	Hellmuth et al. (57)	Perinatal	CTS2	Con	*r* = 0.07, *p* > 0.05
Alcohol misuse	Nathanson et al. (123)	IPV exposed (F)	CTS2	Con	*r* = 0.10, *p* > 0.05
Alcohol misuse	Flanagan et al. (49)	IPV exposed (F)	CTS2/PMWI	Con	*r* = 0.08, *p* > 0.05
Alcohol misuse	Watson-Singleton et al. (99)	IPV exposed (F)	ISA	Con	***r* = 0.19, *p* = 0.020**
Alcohol misuse	Reyes et al. (50)	IPV exposed (F)	PMWI	Con	***r* = 0.34, *p* < 0.01**
Alcohol related problems Alcohol dependence	Sullivan et al. (82)	IPV exposed (F)	PMWI	Con	***r* = 0.38, *p* < 0.01** AOR = 0.98, *p* > 0.05
Deliberate self-harm (DSH)	Jaquier et al. (80)	IPV exposed (F)	PMWI	Con	**Severity of psychological IPV differed significantly between DSH groups, *p* = 0.027, and was highest in the current DSH group**
Suicidal ideation	Kandeğer et al. (7)	IPV exposed (F)	SVAW	Con	***r* = 0.51, *p* < 0.01**
Suicidal behavior	Peltzer et al. (91)	IPV exposed (F)	SVAW	Con - Cat	***r* = 0.33, *p* < 0.01: Symbolic AOR 0.14 (95% CI 0.03, 0.81) Mild 7.11 (95% CI 1.09, 46.43)** Moderate 0.94 (95% CI 0.24, 3.76) Severe 1.79 (95% CI 0.44, 7.18)
CMD: Depressed anxious mood. Depressive thoughts	Santos et al. (68)	Community (F)	CTS2	Cat	Minor OR = 1.42 (95% CI 0.85; 2.36); Severe OR = 1.29 (95% CI 0.76; 2.15) **Minor OR = 2.93 (95% CI 1.72; 4.98); Severe OR = 3.11 (95% CI 1.93; 5.00)**

r, correlation coefficient; b, regression coefficient; AOR, adjusted odds ratio; p, *p*-value; OR, odds ratio; CI, confidence intervals. Variable type, continuous, categorical.

**Table 5:** Association between severity of sexual IPV and mental health outcomes.

MHO	Study	Population F-female, M-male	Tool	Variable type	Findings
Depression	Nathanson et al. (123)	IPV exposed (F)	CTS2	Con	*r* = 0.06, *p* > 0.05
Depression	Sezgin and Punamäki (55)	Perinatal	CTS2	Con	***b* = 0.08, *p* < 0.01**
Depression	Signorelli et al. (58)	Help-seeking (F)	CTS2	Con	***b* = 0.463, *p* < 0.001**
Depression	Esie et al. (61)	Community (F)	CTS2 WHO	Cat	None AOR = 1; Low 0.92 (0.71–1.19); Medium 1.13 (0.86–1.49); **High 1.65 (1.08–2.52)**
Depression	Flanagan et al. (49)	IPV exposed (F)	SES	Con	***r* = 0.28, *p* < 0.01**
Depression	Sullivan et al. (82)	IPV exposed (F)	SES	Con	***r* = 0.29, *p* < 0.05**
Depression	Williams et al. (94)	IPV exposed (F)	SES	Con	*r* = 0.061, *p* > 0.05
Depression	Peltzer et al. (91)	IPV exposed (F)	SVAW	Con	***r* = 0.36, *p* < 0.01: AOR 3.16 (95% CI 1.33, 7.48)**
PTSD	Nathanson et al. (123)	IPV exposed (F)	CTS2	Con	***r* = 0.31, *p* < 0.01**
Trauma symptoms	Yalch et al. (60)	Community (F)	CTS2	Con	***r* = 0.22, *p* < 0.05**
PTSD	Kastello et al. (64)	IPV exposed (F)	CTS2	Cat	No association between categorical severity sexual IPV and PTSD, *p* = 0.958
PTSD	Norwood et al. (52)	Female partners of IPV perpetrators	CTS2 & SES	Con	***r* = 0.25 (total sexual IPV), *p* < 0.01; *r* = 0.21 (sexual coercion and sexual violence), *p* < 0.01**
PTSD	Flanagan et al. (49)	IPV exposed (F)	SES	Con	***r* = 0.39, *p* < 0.01**
PTSD	Sullivan et al. (82)	IPV exposed (F)	SES	Con	***r* = 0.35, *p* < 0.01**
PTSD	Williams et al. (94)	IPV exposed (F)	SES	Con	***r* = 0.186, *p* < 0.01**
PTSD	Sabri et al. (90)	IPV exposed (F)	SVAW		“No significant association was found between sexual abuse and co-occurring PTSD and depression problem”
Anxiety	Sezgin and Punamäki (55)	Perinatal	CTS2	Con	***b* = 0.07, *p* < 0.05**
Drug misuse	Nathanson et al. (123)	IPV exposed (F)	CTS2	Con	*r* = 0.0, *p* > 0.05
Drug misuse	Flanagan et al. (49)	IPV exposed (F)	SES	Con	***r* = 0.22, *p* < 0.01**
Opioids abuse	Williams et al. (94)	IPV exposed (F)	SES	Con	***r* = 0.143, *p* < 0.05**
Drug misuse	Reyes et al. (50)	IPV exposed (F)	SES	Con	***r* = 0.19, *p* < 0.05**
Alcohol misuse	Nathanson et al. (123)	IPV exposed (F)	CTS2	Con	*r* = 0.08, *p* > 0.05
Alcohol misuse	Flanagan et al. (49)	IPV exposed (F)	SES	Con	***r* = 0.25, *p* < 0.01**
Alcohol related problems Alcohol dependence	Sullivan et al. (82)	IPV exposed (F)	SES	Con	***r* = 0.19, *p* < 0.05** AOR = 1.17, *p* > 0.05
Alcohol misuse	Reyes et al. (50)	IPV exposed (F)	SES	Con	***r* = 0.25, *p* < 0.01**
Deliberate self-harm (DSH)	Jaquier et al. (80)	IPV exposed (F)	SES	Con	“Women with current DSH reported greater severity of numbing symptoms and sexual IPV compared to women with past DSH only.” – findings of discriminant function analysis
Suicidal ideation	Kandeğer et al. (7)	IPV exposed (F)	SVAW	Con	***r* = 0.47, *p* < 0.001**
Suicidal behavior	Peltzer et al. (91)	IPV exposed (F)	SVAW	Con	***r* = 0.35, *p* < 0.01**: AOR 2.78 (95% CI 0.88, 8.78)
CMD: Depressed anxious mood. Depressive thoughts	Santos et al. (68)	Community (F)	CTS2	Cat	Minor OR = 1.42 (95% CI 0.64; 3.17); Severe OR = 6.1 (95% CI 0.81; 45.45) **Minor OR = 2.47 (95% CI 1.34; 4.57)**; Severe OR = 2.22 (95% CI 0.94; 5.24)

r, correlation coefficient; b, regression coefficient; AOR, adjusted odds ratio; p, *p*-value; OR, odds ratio; CI, confidence intervals. Variable type, continuous, categorical.

Different statistics were calculated by the statistical analyses, such as correlation coefficients (r) to measure the association between two variables measured on a continuous/discrete scale; regression coefficients (b) used in multiple regression, where the mental health outcome is treated as a continuous variable, and multiple covariates (to account for confounding) are included in the model in addition to IPV variables; and crude odds ratios (OR’s) or adjusted odds ratios (AOR’s) (produced when covariates are included in the model) which were adopted when the mental health outcome was treated as binary, presence of disorder or not, to assess the association between severity of IPV and mental health outcomes.

Twenty-six studies used a measure of severity of *overall* IPV to explore its association with mental health outcomes (Table 2). Depression was the outcome in 17 studies, PTSD/trauma symptoms in 10, anxiety in four, psychological distress in three, and common mental disorder studies, alcohol/opioid abuse, and suicidal ideation/behavior each in two studies.

A small but statistically significant association between the severity of overall IPV and depression was reported in 11 of the 17 studies. However, in the study where they controlled for confounding variables (91), no significant association was found between risk of lethality and depression. In the two studies employing a categorical form of IPV, the OR or AOR are all greater for severe IPV than minor IPV and “very severe” in Mugoya et al. (67). Seven out of the 11 studies reporting a significant association were based on samples of women who had all experienced IPV.

In relation to the association between the severity of combined forms of IPV and PTSD (or trauma symptoms), nine out of 10 studies examining PTSD reported a significant association. Three of the nine studies adjusted for covariates (90, 93, 100) with the latter study reporting a non-significant association after adjustment. These studies mostly were based on samples of women who had all experienced IPV.

Three of the four studies that analyzed anxiety as an outcome found statistically significant associations between overall IPV severity, one of which adjusted for confounding variables (100).

With regards to psychological distress, Tutty et al. (101) reported a small but statistically significant correlation using the CAS total score, while Kamimura et al. (85) found that mean scores of psychological distress did not differ significantly between categories of risk of lethality (as measured by DA). However, in a perinatal study (70) the odds of psychological distress increased in relation to increasing number of different types of IPV.

The two studies examining the use of opioid, and alcohol reported a positive correlation with overall severity of IPV as measured by CTS2. The study by Gibbs et al. (73) examining suicidal ideation found it was more prevalent in women who experienced emotional IPV in combination with physical and sexual IPV, than those with did not report emotional IPV. Whereas Peltzer and Pengpid (91) reported that suicidal behavior was significantly correlated with risk of lethality showing that women in the highest danger category were significantly more likely to exhibit suicidal behavior. Both studies examining common mental health disorders (CMD) reported significant association, with Tran et al. (76) showing that women who had experienced all types of IPV had increased odds of having a CMD, whereas Fisher et al. (72) demonstrated that whether examining lifetime or postpartum IPV the AOR for wo-three types of IPV was greater than that for one type of IPV.

#### 4.2.1 Association between severity of physical IPV and mental health outcomes

Twenty-eight studies adopted a measure of severity of physical IPV to analyze its association with a range of mental health outcomes. Depression was measured in 13 studies, PTSD/trauma symptoms in 10, alcohol/drug abuse in 10, psychological distress in three studies, suicidal ideation/behavior in three, anxiety in two, deliberate self-harm in one, and finally common mental disorders in one study (Table 3).

Eight of the 13 studies measuring depression used the CTS2 to measure severity of physical IPV. Depression was significantly associated with the severity of physical IPV in nine studies. Four of the five studies reporting statistically significant correlations presented coefficients from 0.22 to 0.355. However, in one study (91) when the categorical forms of IPV severity and depression were used and covariates were adjusted for, the AOR’s were not significant. Further, the study by Esie et al. (61) using a categorical form of severity indicated that women experiencing medium or high severity of physical IPV had increased odds of being depressed. In Lobato et al. (65) a significant association between severity of physical IPV and post-natal depression was highlighted, which appeared to be dependent on whether the partner misused alcohol or not. A study set in the community using a categorical form of SVAW (67) showed that while both minor and severe physical IPV were associated with greater odds of depression the AOR for severe was greater than for minor. In the study by Xu et al. (79) regression analysis indicated a significant association between severity of physical IPV and depression for both men and women.

Most of the studies examining the severity of physical IPV and its association with PTSD/trauma symptoms used the CTS2. Five of these seven studies reported a significant association with correlation coefficients ranging from 0.25 to 0.54. A high, statistically significant correlation (*r* = 0.719) between severity of physical IPV as measured by CAS and PTSD was highlighted in a sample of women who had all experienced IPV (96). The study by Sabri et al. (90) used a composite outcome of PTSD and depression and reported greater severity of physical IPV in women with both PTSD and depression than those with depression alone.

Both studies examining anxiety indicated that the severity of physical IPV was significantly associated with anxiety. In Kita et al. (98) they adopted the ISA to assess anxiety in the antenatal and postnatal periods, respectively *r* = 0.12 and 0.14, whereas Wadji et al. (102) found a statistically significant correlation (*r* = 0.43.) between severity of physical IPV and anxiety using CTS2.

With regards to psychological distress, the two papers by Hellemans et al. (77, 78) reported small correlations between physical IPV severity and psychological distress, though only Hellemans et al. (78) achieved statistical significance with *r* = 0.17. In Kaplan et al. (63) the authors reported two analyses, the first of baseline psychological distress, the second of change in psychological distress over 2 years. They used a categorical form of CTS2 with minor and severe physical IPV variables entered into multiple regression models alongside covariates. Regression coefficients were small with only the minor severity of physical IPV being associated with baseline psychological distress. In a perinatal study (70) using SVAW, the authors reported statistically significant associations between moderate and severe physical IPV and psychological distress, AOR’s of 2.41 and 3.25, respectively.

Most of the studies examining alcohol or drug misuse as the outcome used the CTS2 to measure severity of physical IPV. Table 2 shows statistically significant correlation coefficients being reported in six studies ranging in magnitude from 0.14 to 0.41. Moreover, of the four studies examining suicidal behavior/self-harm two studies found statistically significant correlations (7, 91), though in the latter further analysis incorporating covariates showed non-significant associations between increasing severity categories of physical IPV and outcome. Finally, Santos and Monteiro (68) examined common mental disorders and were able to show that while minor physical IPV was significantly associated with increased odds of having a depressed anxious mood, severe physical IPV was not. They also showed that while both minor and severe physical IPV was associated with greater odds of depressive thoughts, the AOR for severe was greater than for minor.

#### 4.2.2 Association between severity of psychological IPV and mental health outcomes

Illustrated in Table 4 are the 24 studies that conducted 42 analyses examining the association between severity of psychological IPV and a range of mental health outcomes: depression (12 studies), alcohol/drug abuse (nine), PTSD/trauma symptoms (eight), anxiety (two), psychological distress (two), suicidal ideation/behavior (two), and deliberate self-harm and common mental disorders (CMD) (one study each).

Overall, severity of psychological IPV was significantly associated with depression in 10 studies (11 analyses, highlighted in bold in Table 4). Seven of these analyses reported correlation coefficients from 0.18 to 0.46, all *p* < 0.01. The studies by Mugoya et al. (67) and Esie et al. (61) showed significant associations between severity of psychological IPV and depression but only at the “severe” rating of IPV. By contrast the study by Peltzer and Pengpid (91) that used the categorical form of the SVAW measure in subsequent regression analysis, did not find a statistically significant association. In Tho Tran et al. (75) they found increasing AOR’s with increasing number of types of emotional violence (expressed categorically). The lower confidence intervals around these AOR’s are above one except for the highest number of types of emotional violence category which encompasses one. The study by Xu et al. (79) reported significant associations between severity of psychological IPV and depression in both male and female participants.

Table 4 indicates that the severity of psychological IPV and PTSD are significantly associated in seven studies, with statistically significant correlation coefficients being reported in five studies ranging from 0.22 to 0.56. In the two studies examining anxiety as the outcome, only Kita et al. (98) found statistically significant correlation coefficients between severity of psychological IPV, as measured by ISA, and anxiety in both the ante and postnatal periods, *r* = 0.22 and 0.24, respectively. Moreover, both studies by Hellemans et al. (77, 78) which examined the association between psychological distress and severity of psychological IPV, reported statistically significant correlations of 0.19. However, the sample of adults of Turkish origin in Hellemans et al. (77) is a subsample (*n* = 392) of the general population sample in the other study by the same author (*n* = 1,445).

In the 10 analyses examining the association between severity of psychological IPV and alcohol/drug abuse, four showed a statistically significant correlation of severity of psychological IPV and use of substances. In Flanagan et al. (49) severity of psychological IPV was statistically significantly correlated (*r* = 0.11) to drug use, while correlations ranged from 0.19 to 0.38 in three studies (50, 82, 99) examining the association between the severity of psychological IPV and alcohol use. However, Sullivan et al. (82) also found that severity of psychological IPV was not a predictor of alcohol dependence in a regression analysis controlling for covariates.

The three studies exploring the association between severity of psychological IPV and suicide reported statistically significant positive correlations. However, Peltzer and Pengpid (91) conducted a regression analysis which did not provide evidence of a significant relationship between moderate and more severe IPV and greater odds of suicidal ideation/behavior. Finally, Jaquier et al. (80) indicated that severity of psychological IPV differed between three groups of women: those who currently self-harm, those who had in the past, and those who had never done so. Women who currently self-harm had the highest mean score of severity of psychological IPV. In the study by Santos and Monteiro (68) examining common mental health disorders, they reported significant associations between severity of psychological IPV and depressive thoughts at both minor and severe ratings of IPV. In this same study, depressed anxious mood was not associated with either minor or severe IPV.

#### 4.2.3 Association between severity of sexual IPV and mental health outcomes

In Table 5 we highlight the 17 studies that explored the association between severity of sexual IPV and mental health outcomes: alcohol/drug use (nine studies), PTSD/trauma symptoms (eight studies), depression (eight studies), suicidal ideation/behavior (two studies), anxiety (one study), deliberate self-harm (one study) and common mental health disorders (one study).

Of the eight analyses of depression, six had significant associations between the severity of sexual violence and depression, three of which found correlation coefficients ranging from 0.29 to 0.36. Moreover, Esie et al. (61), where they used a four-level categorical rating of severity of sexual IPV, they found that just the highest severity of sexual IPV was statistically significantly associated with depression with AOR equal to 1.65. In studies using regression analyses (55, 58) significant associations between severity of sexual IPV and depression remained after multiple regression. The study by Peltzer and Pengpid (91) reported statistically significant associations between severity of sexual IPV and depression when depression was analyzed as both continuous (with correlation) and dichotomous (with logistic regression).

Of the eight studies examining the association between the severity of sexual IPV and PTSD, six reported statistically significant correlation coefficients ranging from 0.186 to 0.39. The study by Sezgin and Punamäki (55) using multiple regression models reported a significant positive association between severity of sexual IPV and anxiety. Moreover, six of the nine studies examining use of drugs or alcohol as an outcome, reported statistically significant correlation coefficients ranging from 0.143 to 0.25.

Suicidality was analyzed as the outcome in three studies, two of which reported statistically significant correlations between severity of sexual IPV and suicidal ideation/behavior, *r* = 0.35 and 0.47. However, Peltzer and Pengpid (91) went on to explore the association further in a logistic regression model and reported a non-significant AOR. Jaquier et al. (80) found that severity of sexual IPV significantly discriminated between women who currently self-harm and those who have done in the past, with those who currently self-harm scoring higher on severity of sexual IPV. An unadjusted analysis by Santos and Monteiro (68) found that minor severity of IPV was statistically significantly associated with depressive thoughts, while severe IPV was not.

## 5 Discussion

This review, comprising 76 studies, identified 22 measures utilized to evaluate the prevalence, incidence, risk, and severity of IPV and its association with mental health outcomes. The review underscored researchers’ inclinations to modify IPV measures frequently without reassessing their validity. Additionally, the commonly used measure CTS2 was seldom applied in its initially validated form. By contrast, measures exclusively measuring a single subtype of IPV, especially those developed more recently, were rarely modified. We found inconsistent findings regarding minor and severe categorical ratings of IPV severity. The examination of evidence concerning the correlation between the severity of IPV and mental health outcomes emphasizes the need for the application of statistical methods that produce more robust and accurate estimates of effect. Particularly, these estimates should be adjusted for relevant confounding variables using regression models to reduce bias.

### 5.1 Measurement of IPV severity in practice

Previous research has assessed the psychometric properties of IPV measures [see (103–105)]. In our review we found that numerous studies altered the measures of IPV severity. This raises concerns about the potential impacts on the psychometric properties of the measures and in so doing jeopardizes the credibility and applicability of research findings in this critical area. It is important that researchers scientifically demonstrate the quality of their methods of measurement by showing that they are statistically reliable (13) whereby indicating how consistently the new construct is measured (e.g., *test–retest reliability, internal reliability*). Undertaking appropriate validity tests (e.g., *content validity, construct validity, predictive validity*) is key to being confident that the data, as collected and analyzed, accurately capture the true picture of what is being measured.

The operationalization of violence severity has also varied across studies: we identified scoring inconsistencies which compromised the assurance that the severity levels assigned to various incidents held uniform meaning and implications across studies. Two types of categorical ratings for the severity of IPV were found: the severity classifications were either determined by (1) the creators of assessment tools (e.g., CTS2 and WHO tools), who categorized acts as “minor” or “severe,” or (2) by the authors of individual studies. For instance, Esie et al. (61) established categories (low, medium, high) based on cut-off points from the continuous form of IPV severity, while Mugoya et al. (67), used the number of types of IPV experienced for their categories. Lack of consistency in applying validated methods for scoring instruments to determine abuse severity may reflect the lack of consensus in defining abuse (13). When making scoring decisions researchers face real difficulties in establishing reasonable comparison groups to investigate differences that might inform interventions.

Another concern arises from the practice of categorizing incidents at a single point in time, in cross-sectional studies, which would not accurately capture changes in the severity of IPV over time. This approach could overlook the escalation or de-escalation of violence and result in underreporting. Survivors may be reluctant to report incidents, especially when a relationship has not been established with researchers, due to fear or shame. The use of categorical measures may contribute to underreporting, as survivors might only disclose incidents they perceive as “severe,” potentially neglecting less severe occurrences. This selective reporting, combined with the normalization of IPV in societies (2), can lead to an inaccurate portrayal of the prevalence and distribution of IPV. This is important because underreporting means that services and support cannot be put in place. Research shows (106) that individuals report minor physical violence on measures such as the CTS, but do not report such assaults on crime victimization scales or when asked a general question about experiencing physical violence in a relationship because they usually do not interpret such aggression as having the significance of a legally defined assault. In their study Hamby et al. (106) compared endorsement of the CTS’s physical aggression items with subjective reports of experiencing partner violence, and found that minor and infrequent moderate acts of physical aggression that were endorsed on the CTS were not reported as subjective experiences of partner aggression.

Creating categorical ratings of IPV severity from continuous scores may simplify analysis and interpretation, but it also comes with several limitations. These include: (i) loss of information which can produce less accurate and precise results and therefore a reduction in statistical power, (ii) arbitrary cut-off points meaning that results are not reproducible across studies, and (iii) misrepresented relationships between variables, where arbitrary cut-off points mean that the nuances of the original variable distribution are no longer present (107). In contrast, the practice of dichotomizing the sample by categorizing individuals into two groups for analysis—such as placing anyone who has encountered at least one instance of IPV into the abuse group and categorizing everyone else with zero occurrences in each category into the non-abused group—is misleading (13). This dichotomous classification for victimization combines individuals who have experienced a single incident with those who have undergone extensive victimization. Research studies have shown that individuals experiencing very small amounts of IPV generally appear to be much more similar to those experiencing no IPV behaviors (108). Therefore, dichotomization solely based on the experience of any IPV is prone to misinterpretation. There is a risk of overlooking effects linked to a higher threshold of abuse within a relationship when individuals surpassing that threshold are grouped together with those who have encountered minimal IPV, resulting in an averaging effect.

### 5.2 The association between severity of IPV and mental health outcomes

A number of studies showed that increasing severity of IPV, when measured using “minor” and “severe” categorizations of IPV, was significantly associated with poorer mental health (see Tables 2, 3) (67, 70). At the same time, other studies reported that ‘minor’ or lower severity of IPV was not linked to poorer mental health, but when the violence was more severe, mental health tended to suffer (see Tables 3, 4) (61, 67). However, our review also revealed examples of statistically significant associations between minor IPV and outcome, with severe IPV and outcome unrelated, despite higher adjusted odds ratios (AOR) in Peltzer and Pengpid (91) and Santos and Monteiro (68) studies. These apparent false negatives may occur because severe IPV is less common and therefore the parameter estimates are less precise, increasing the risk of a Type II error.

In our review, the severity of IPV and its subtypes was consistently linked to various mental health outcomes across studies. We identified evidence that experiencing more subtypes of IPV was associated with poorer mental health outcomes (70, 72, 76, 109). Generally, more severe overall IPV and its subtypes correlated with poorer mental health outcomes, as indicated by positive correlation and regression coefficients, and Odds Ratios (ORs) and AORs greater than 1. Our analyses did not reveal wholly consistent patterns that would allow for a comprehensive determination of how distinct IPV subtypes affect mental health outcomes differently, but we speculate that the mental health outcome most affected by increasing severity of physical IPV is PTSD. Increasing severity of psychological IPV appears to be most constantly associated with depression. Severity of sexual IPV was explored in less studies but the evidence of its impact varying dependent on mental health outcomes was less compelling. While ideal, conducting meta-analyses to establish robust pooled estimates of these relationships faces challenges due to significant clinical and statistical heterogeneity, especially considering variations and inconsistencies in measuring and analyzing IPV severity across studies (1). Performing meta-analyses to unpick the impact of differing severity within subtypes of IPV is unlikely to produce valid and reliable results.

The studies reviewed exhibited variation in the assessment of mental health outcomes. Some studies evaluate mental health on a spectrum, while others use a dichotomous approach. These differing methods pose distinct questions: does increased severity of IPV correlate with more pronounced mental health symptoms, or does heightened severity of IPV increase the likelihood of exceeding the threshold indicative of clinically significant mental health outcomes? This variability is influenced by the study population, as some studies recruit participants based on clinical diagnoses.

Studies in the review differed with regards to the populations being studied and we categorized them as those which focused on women with previous IPV experiences, those in the community and those in perinatal samples. Without a prerequisite of IPV exposure, any measure of IPV severity showed zero-inflation, indicating that a significant proportion of the sample had not experienced IPV. This resulted in highly skewed severity scores (59, 63, 67, 69), posing challenges to analysis and interpretation, such as violating statistical assumptions and lacking sensitivity in modeling the true relationship. To address skewness, some studies applied transformations (59), though these could not correct for zero-inflation. Others (63, 67, 69) accounted for zero-inflation by using categorical forms of IPV severity; these have their own limitations as illustrated earlier.

In evaluating the association between IPV severity and mental health outcomes, it is crucial to critically assess the statistical analyses employed in these studies. Many studies relied on correlation coefficients. However, correlation coefficients are valid only for linear relationships between two variables and may oversimplify the complex connections between IPV and mental health, potentially missing nonlinear or threshold effects. Statistically, correlation coefficients measure the strength of a linear relationship along a continuous scale, but their interpretation can be misleading (110). Significance tests may yield statistically significant results with large sample sizes, even when the correlation value is clinically irrelevant. Statistical literature emphasizes the cautious interpretation of correlation coefficients (111–113). These coefficients are inadequate for determining causality direction—whether IPV directly causes mental health outcomes, vice versa, or if other factors influence both variables. Many reported coefficients serve as a preliminary analysis, preceding more comprehensive methods like structural equation modeling. Correlation coefficients alone are insufficient to describe the relationship and do not consider potential confounding variables such as socioeconomic status, social support, trauma history, responses to disclosures, and access to mental health resources. Regression models were used by some studies [e.g., (54, 55)] allowing the inclusion of potentially confounding variables into the model. These models can be extended for longitudinal studies which can support claims of temporal causality.

Another issue is the lack of survivor involvement in the development, scoring and weighting of IPV measures. Of the eight commonly used IPV measures, only one explicitly involved people with lived experience of IPV in their development, and none reported involving people with IPV in decisions about scoring and weighting. This was the Danger Assessment Scale which was developed with consultation and content validity support from IPV survivors, shelter workers, law enforcement officials, and other clinical experts on IPV. In addition, the WHO Multi-country Study on Women’s Health and Domestic Violence Against Women had an expert consultation group on violence against women bringing together researchers, health care providers and women’s health advocates from several countries. The lack of survivor involvement might impact the ecological validity of the measures - their ability to reflect the real world (114). This could minimize or inflate the severity and impact of IPV incidents, bearing in mind their complexity and location in dynamic and evolving circumstances. There is also a risk that where measures are self-report (n = 6), researchers assume they are hearing directly from people who have experienced IPV and are capturing issues that are important and relevant to them. However, as the measures themselves might not reflect how people with lived experience understand, experience and weight the severity of IPV incidents, the information gathered is likely to be partial, potentially only capturing *researcher’s* conceptualizations of IPV severity. This raises the possibility of confirmation bias.

Finally, to the best of our knowledge, none of the eight IPV measures assessed the acceptability of the content to people who have experienced IPV. Acceptability, defined as a subjective evaluation of an intervention’s content made by their recipients, is important because successful implementation depends on the acceptability of the intervention to recipients and needs to be considered in the development, evaluation and implementation phases of any healthcare interventions (115). Completion of measurement tools can be considered a healthcare intervention particularly when being used in routine clinical practice. Acceptability is a precursor to fidelity (use as intended) which is a precursor for implementation (116). In reviewing measures, we noted that questions are deeply intrusive by their nature, and potentially distressing and shaming. This, coupled with the victim-blaming that is present across societies, could result in significant under-reporting as well as minimization of the severity of incidents and a lack of acceptability to users. We must ask ourselves what it is that measures of IPV severity are able to reveal.

### 5.3 Limitations

Undertaking secondary data analysis research avoids study repetition and over-research of sensitive topics/populations. However, there are drawbacks of utilizing data from a previous systematic review. For instance, the last search was conducted a considerable time ago (November 2020), potentially missing out on pertinent studies related to the topic. However, recent papers are unlikely to alter the established findings on the severity of IPV and its impact on mental health outcomes. Additionally, the eligibility criteria for the systematic review may not be optimal for addressing the current research question. In addition to this the limitations in the included studies, such as the researchers’ practice of deviating from the original scoring scheme of the IPV severity measures, made it impossible for us to directly compare findings across different studies or contexts. The heterogeneity of the included studies (e.g., diverse populations, settings, measurement tools and participant characteristics) was a challenge as we could not consider pooling data for secondary analysis which could have enhanced the generalizability and interpretation of the findings. The absence of standardized reporting for results and outcomes also presented a difficulty, as inconsistent reporting standards impeded our ability to effectively synthesize findings across studies. Furthermore, another limitation is that we did not reach out to authors to obtain any missing data.

### 5.4 Recommendations

When assessing incidents of IPV we recommend adopting a dynamic and longitudinal approach. Rather than categorizing incidents at a single point in time, practitioners should consider implementing methods that allow for the monitoring and evaluation of changes in the severity of IPV over time. This may involve utilizing measures or assessments that capture the evolving nature of IPV experiences and patterns, providing a more accurate and comprehensive understanding of the dynamics involved. Longitudinal assessments can contribute to a more nuanced and contextually rich perspective, enabling interventions and support services to be tailored to the evolving needs of individuals experiencing IPV.

Considering the outcomes of our review, which revealed the inadequacy of existing measures in assessing IPV and its severity, we propose the development of a new measure, one that actively involves individuals with lived experiences of IPV in the development, scoring, and weighting processes. The aim would be to create a measure that is not only scientifically rigorous but also ethically and culturally appropriate, promoting a more comprehensive and empathic understanding of IPV. Ample evidence exists of methods to generate reliable and valid outcome measures from the perspectives of service users (117, 118); these could be adopted by researchers working with IPV survivors. The model involves participatory qualitative and psychometric methodology to explore survivors’ experiences and perspectives and translate these into psychometrically robust outcome measures (119).

Addressing cross-cultural considerations in the measurement of IPV is crucial because how IPV is understood within a particular culture can significantly impact its identification, risk assessment, and connection to care. Cultural norms may influence what can be measured in research or clinical settings. For instance, cultural sanctions might restrict the disclosure of sexual IPV, limiting the ability to measure its effects on mental and physical health or its inclusion as an outcome in interventions (109, 120, 121). Additionally, these norms can shape how questions are framed, affecting the translation and adaptation of assessment tools across different regions.

Moreover, enhancing coordination and collaboration across sectors in the collection of IPV data is essential, as various agencies—such as health services, specialist services, criminal justice, and welfare services—must work together to reduce and eliminate violence (15). It is also important for researchers and policymakers to collect data that aligns with their specific areas of responsibility. Definitions and interpretations of IPV vary between and within disciplines and sectors. While some of this variation reflects the differing priorities of these agencies, which is often justified, other differences are simply historical and offer little practical value. Even when complete alignment in the conceptualization and measurement of violence across fields is not possible, the frameworks should at least be compatible or translatable (2).

## 6 Conclusion

There is a tendency in many research studies of intimate partner violence to inadequately characterize the distribution of severity of violence in the study sample, crucially impacting on our ability to interpret results and making meaningful comparisons across studies. IPV is multifaceted, with acts and forms that can shift and overlap, creating dynamic and concurrent patterns. This complexity poses significant challenges for measurement, as it requires capturing not just individual instances but also the evolving and interacting nature of violent behaviors. Traditional measurement tools may struggle to account for these fluid dynamics, making comprehensive assessment more difficult. However, accurate measurement is essential for assessment of the relationship between severity of IPV and mental health problems, one that is developed with and acceptable to individuals with experience of IPV. Men and women exposed to a range of types and severity of IPV can experience a broad spectrum of adverse mental health outcomes. However, it is not possible to make more definitive, specific claims regarding the relative effects of IPV subtypes on mental health. Chronic exposure to IPV is associated with heightened mental health issues, although this association is influenced, at least in part, by the specific type of IPV encountered.

## Data Availability

The original contributions presented in the study are included in the article/supplementary material, further inquiries can be directed to the corresponding author.
